# The Effect of Pyrroloquinoline Quinone on the Expression of WISP1 in Traumatic Brain Injury

**DOI:** 10.1155/2017/4782820

**Published:** 2017-08-16

**Authors:** Yongqi Ye, Pengju Zhang, Yuhang Qian, Baoxin Yin, Meijuan Yan

**Affiliations:** The Jiangsu Key Laboratory of Neuroregeneration, Nantong University, 19 Qixiu Road, Nantong 226001, China

## Abstract

WISP1, as a member of the CCN4 protein family, has cell protective effects of promoting cell proliferation and inhibiting cell apoptosis. Although some studies have confirmed that WISP1 is concerned with colon cancer and lung cancer, there is little report about the influence of WISP1 in traumatic brain injury. Here, we found that the expression of WISP1 mRNA and protein decreased at 3 d and then increased at 5 d after traumatic brain injury (TBI). Meanwhile, immunofluorescence demonstrated that there was little colocation of WISP1 with GFAP, Iba1, and WISP1 colocalized with NeuN partly. WISP1 colocalized with LC3, but there was little of colocation about WISP1 with cleaved caspase-3. Subsequent study displayed that the expression of *β*-catenin protein was identical to that of WISP1 after TBI. WISP1 was mainly located in cytoplasm of PC12 or SHSY5Y cells. Compared with the negative control group, WISP1 expression reduced obviously in SHSY5Y cells transfected with WISP1 si-RNA. CCK-8 assay showed that pyrroloquinoline quinone (PQQ) had little influence on viability of PC12 and SHSY5Y cells. These results suggested that WISP1 played a protective role after traumatic brain injury in rats, and this effect might be relative to autophagy caused by traumatic brain injury.

## 1. Introduction

Traumatic brain injury (TBI), also known as brain injury, is mainly caused by external mechanical forces. There were a series of pathological, physiological, and biochemical changes, such as subarachnoid hemorrhage, cerebral blood tube spasms, disturbance of cerebral circulation, and cerebral edema. All these secondary and primary brain injuries led to higher mortality rates. In the present study, Feeney et al.'s [1] method was used to establish a traumatic brain injury (TBI) model in rats.

WISP1 (Wnt1 inducible signaling pathway protein 1) is a CCN family member, which is more broadly identified with development and tumorigenesis [2]. CCN protein family consists of 6 family members, including cysteine-rich protein 61 (CYR61/CNN1) and connective tissue growth factor (CTGF/CCN2), as well as nephroblastoma-overexpressed secreted protein (NOV/CCN3), WISP1 (CCN4), WISP2 (CCN5), and WISP3 (CCN6) [3]. The CCN family is characterized by four cysteine-rich modular domains that include insulin-like growth factor-binding domain, von Willebrand factor type C module, thrombospondin domain, and C-terminal cysteine knot-like domain. In the extracellular matrix, WISP1 combinates leucine-rich proteoglycans and affects ability of the cell to anchor the extracellular matrix [4].

WISP1 could express in a variety of tissues, and there is no tissue specialty. Pennica et al. discovered that WISP1 was expressed in many tissues including the adult heart, lung, kidney, small intestine, spleen, pancreas, ovaries, and brain, but there were significance changes about express levels among different tissues [5]. WISP1 could block p53-mediated DNA damage and apoptosis [6] and promote cell proliferation and cell adhesion [7]. WISP1 was associated with neoplastic growth [8]. WISP1 has a relationship with the formation and evolution of lung cancer, renal cell carcinoma, colorectal cancer, and other tumors. In the recent years, study showed that WISP1 could promote cardiac remodeling following myocardial infarction [7] and lung tissue repair and regrowth [9]. In addition, WISP1 may play a vital role in bone formation and fracture repair [10, 11] and can limit neuronal cell injury during oxidative stress [12].

*β*-Catenin is a soluble protein located in the cytoplasm which is first found in 1980 by German scientists [13]. Subsequent studies show that *β*-catenin is homology analogs of armadillo gene of Drosophila in mammals [14]. Intracellular *β*-catenin mainly exists in the cell membrane, cytoplasm, or nucleus in complex forms. The location of *β*-catenin in the cell is related to biological functions. For *β*-catenin in cell membrane, *β*-catenin protein in normal mature cells mediated cell adhesion and migration and affected the polarity and integrity of the epithelium [15]. *β*-Catenin in the cytoplasm passes through into the nuclear, leading to gene transcription, which is closely related to the development and progression of many diseases [16]. *β*-Catenin in the nucleus promotes transcription of downstream target genes, which accelerate the cell cycle, promote cell proliferation, produce abnormal protein, and eventually lead to the occurrence of tumor [17].

WISP1, as a CCN family member, utilizes protective pathways that include the traditional wingless canonical and noncanonical signaling of Wnt1. In addition, WISP1 can increase the nuclear expression of *β*-catenin [4]. Interestingly, study finds that *β*-catenin can promote the expression of WISP1 by Wnt1 signaling [18]. WISP1 and its signaling pathways with *β*-catenin represent a novel target that has the potential ability to promote tissue proliferation, repair, and regeneration in multiple cell systems [4, 19, 20].

Pyrroloquinoline quinone (PQQ) is a new oxidoreductase coenzyme which was discovered in the late 1970 of the 20th century. And it is an anionic water soluble compound which exists in almost all biological tissues [21]. PQQ has also been attended broadly in nutrition and pharmacology as an important antioxidant or nutrient. Numerous studies have shown that PQQ has many other pharmacological effects, such as anti-inflammatory and liver and heart protection [22]. It is reported that PQQ-deficient diet cause impaired growth, immunological defects, and decreased fertility in mice [23].

Recent studies have shown that PQQ alters intracellular signaling pathways. For example, when the phosphatidylinositol-3-kinase (PI3K)/AKT signaling pathways are blocked, the protective effects of PQQ will be lost, which implicates that PQQ could regulate neuronal cell survival by (PI3K)/AKT signaling pathway [24]. Similarly, WISP1 has been shown to rely on PI3K and AKT to provide cytoprotection in neurons [4, 12]. WISP1 ultimately modulates apoptotic pathways of Bad, glycogen synthase kinase-3*β* (GSK-3*β*), Bim, Bcl-xL, mitochondrial membrane permeability, cytochrome c release, and caspase activation to prevent cell injury [12]. PI3K and AKT are critical pathways to foster cellular proliferation and block apoptotic injury. However, whether WISP1 can be affected by the activation of PQQ by PI3K/AKT has not been reported.

Although many experiments have confirmed that WISP1 has obvious effective on tissue proliferation, repair, and regeneration, there is little report about the effect of WISP1 in traumatic brain injury. In the present study, we try to explore the effect of WISP1 protein on cell expression, which is necessary to treat brain injury by understanding brain injury repair processes. Therefore, we have established a rat model of TBI to try to further research the function of WISP1 in traumatic brain injury.

## 2. Materials and Methods

### 2.1. Animals and the TBI Model

Sprague-Dawley (SD) rats (200–250 g) were obtained from the Experimental Animal Center of Nantong University (Nantong, China). All animals (*n* = 117) were divided into thirteen groups: sham, 1 d, 3 d, 5 d, and 7 d post-TBI and 1 d, 3 d, 5 d, and 7 d post-TBI + 1 mM PQQ or 2 mM PQQ. Referring to Feeney et al.'s [1] TBI method model, briefly, the rats were deeply anesthetized with chloral hydrate (10% solution), and the heads were fixed in the stereotactic frame, and a 10 mm diameter craniotomy were performed adjacent to the central suture, midway between the lambda and the bregma. The dura was kept intact over the cortex. Injury was delivered by impacting the right cortex with a fluid percussion brain injury device (AmScien Instruments, Richmond, USA). Sham rats (*n* = 9) were craniotomized only. After all the procedures, animals were returned to their cages and allowed freely to get the food and water. Animals were housed under a 12 h light/dark cycle, and room temperature was kept at 25 ± 0.5°C.

### 2.2. Real-Time PCR Analysis

Total RNA was extracted from the frozen cortex brain tissues with TRIZOL Reagent (Sigma) according to the manufacturer's recommendations. The RNA was reverse-transcribed to cDNA using reverse transcription kit (Thermo) with oligo(dT)_18_ primers. Primers of WISP1 and GAPDH were designed with primer 5 software and synthesized by biotech company (GENEray, Shanghai, China), WISP1 sense primer: 5′-GCCCGAGGTACGCAATAGGAGT-3′ and antisense primer: 5′-CCCACGGTGCCATCAATACAGG-3′; GAPDH sense primer was also designed in same way, GAPDH sense primer: 5′-CAACGGGAAACCCATCACCA-3′ and antisense primer: 5′-ACGCCAGTAGACTCCACGACAT-3′. Quantitative real-time PCR analysis was performed using the LightCycler 96 (Roche), applying real-time SYBR Green PCR technology (Roche). The reaction mixtures contained 5 *μ*l SYBR Master Mix, 0.1 *μ*l of each PCR forward and reverse primer (10 *μ*M), 1 *μ*l cDNA, and 3.9 *μ*l nuclease-free water for a final volume of 10 *μ*l. After one cycle of 95°C for 10 min, 45 PCR cycles were performed, each consists of a denaturation step (95°C, 10 s) and an annealing step (60°C, 30 s). Total RNA concentrations from each sample were normalized by the quantity of GAPDH mRNA. All experiments were repeated at least three times.

### 2.3. Western Blot

The brain tissues were stored in −80°C; tissue samples were lysed with extraction buffer. Concentration of the protein was tested using bicinchoninic acid assay kit (Beyotime, Jiangsu, China), followed by electrophoresis separation on SDS-PAGE, and then transferred to a PVDF membrane (Millipore, Bedford, MA, USA) for 120 min at 100 V. The membranes were blocked with 5% skim milk and then incubated overnight with WISP1 antibody (diluted 1 : 500 in TBS; Santa Cruz, Cell Signaling Technology), *β*-actin (1 : 4000, Sigma), and *β*-catenin (1 : 500, Santa Cruz). The PVDF membrane was washed with TBST (TBS with 0.1% Tween 20) for 10 min at least three times and incubated with corresponding HRP-conjugated secondary antibody for 2 h at room temperature. After washing the PVDF membrane for 10 min at least 3 times, the protein was visualized using Beyo ECL Star (Beyotime, Jiangsu, China).

### 2.4. Immunofluorescence

The brain tissue was fixed with 4% paraformaldehyde at 4°C, after the brain tissues sink to the bottom of the bottle and after dehydrating by concentration of gradient (10%, 20%, 30%, and 5%), and then, 12 *μ*m frozen sections were prepared and examined. All sections were blocked with blocking solution (10% goat serum, 3% BSA, and 0.1% Triton X-100) for 1 h at 37°C, incubating overnight with antibody WISP1 (diluted 1 : 500; Santa Cruz), anti-GFAP (1 : 400; BD Pharmingen), anti-NeuN (1 : 1000; Abcam), *β*-catenin (1 : 500; Santa Cruz), anti-cleaved caspase-3 (1 : 400; Cell Signaling Technology), anti-LC3 (1 : 500; Cell Signaling Technology), and anti-Iba1 (1 : 1000; Wako) and then washing them with 0.01 M PBS for 10 min at 3 times and followed by incubating with a mixture of FITC- or Cy3-conjugated secondary antibodies for 2 h at room temperature and then being washed again in PBS for 10 min at 3 times. The stained sections were examined with a Leica fluorescence microscope (Leica DM 5000B, Germany).

### 2.5. EdU Assay

Cell proliferation was detected by Cell-Light EdU DNA cell proliferation kit (RiboBio, Guangzhou, China) according to the manufacturer's instructions. Astrocytes were cultured into 96-well plates at 4 × 10^4^ cells/well. After being incubated with 50 *μ*M EdU for 24 h, cells were washed with PBS, followed by fixation in 4% formaldehyde for 30 min, then being permeabilizated in 0.5% Triton X-100 for 10 min. After extensive washing with PBS, cells were incubated with Apollo for 30 min and Hoechst for 30 min. Proliferative cells was calculated as the percentage of EdU-positive cells relative to the total number of cells.

### 2.6. Transwell Migration Assay

Astrocytes were cultured at 4 × 10^3^ cells/well with DMEM in the upper chamber of a 24-well transwell chamber with 8 *μ*m pore size polycarbonate filters (Costar). 10% fibronectin was added in the lower chamber as chemoattractant. Astrocytes migrate for 24 h and then followed by fixation in 4% formaldehyde for 1 h and stained with 0.1% crystal violet. Images were taken with a microscope and five fields were counted.

### 2.7. Cell Culture

The SHSY5Y and PC12 cells were obtained from the Chinese Academy of Sciences (Shanghai, China). The cells were cultured in DMEM with 10% FBS at 37°C in an incubator containing 5% CO_2_.

### 2.8. Transfection

To silence the expression of WISP1 gene, WISP1 si-RNA was obtained (Invitrogen), WISP1 sense primer: 5′-GGACAUCCAUACACUCAUUTT-3′ and antisense primer: 5′-AAUGAGUGUAUGGAUGUCCTT-3′. si-RNA transfection was performed with lipofectamine 3000 (Invitrogen) according to the manufacturer's guidelines. All data were obtained after being transfected for 72 h.

### 2.9. CCK-8 Test

SHSY5Y cells and PC12 cells were seeded onto 96-well plate ahead of time and then treated with different concentrations of PQQ for 24 h. Subsequently, cells were incubated in a humidified atmosphere with 5% CO_2_ at 37°C for 2 h after adding CCK-8 test solution (Dojindo, Japan), and the cell viability were detected by using microplate reader at 450 nm.

### 2.10. Statistical Analysis

For each experiment, the mean and standard error were determined. The data were analyzed by means of analysis of variance (one-way ANOVA). Statistical significance was determined at the level of *P* < 0.05.

## 3. Results

### 3.1. Expression and Localization of WISP1 after TBI

**Figure 1:**
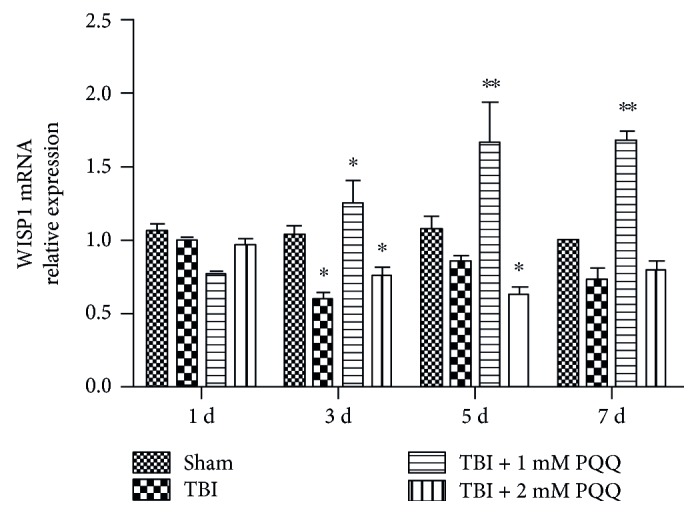
The expression of WISP1 mRNA after TBI (^∗^*P* < 0.05, ^∗∗^*I* < 0.01 versus sham). 1 d: ipsilateral 1 d post-TBI group; 3 d: ipsilateral 3 d post-TBI group; 5 d: ipsilateral 5 d post-TBI group; 7 d: ipsilateral 7 d post-TBI group.

In order to observe the WISP1 mRNA expression after TBI, qRT-PCR was performed. The results showed that, compared with the sham group, WISP1 mRNA slightly decreased at 1 d, reached the minimum at 3 d after TBI, and then recovered at 5 d and 7 d after TBI. Meanwhile, 1 mM or 2 mM PQQ increased WISP1 mRNA expression at 3 d, 5 d, and 7 d post-TBI, but the effect of 1 mM PQQ was obvious to the 2 mM PQQ (Figure 1).

**Figure 2:**
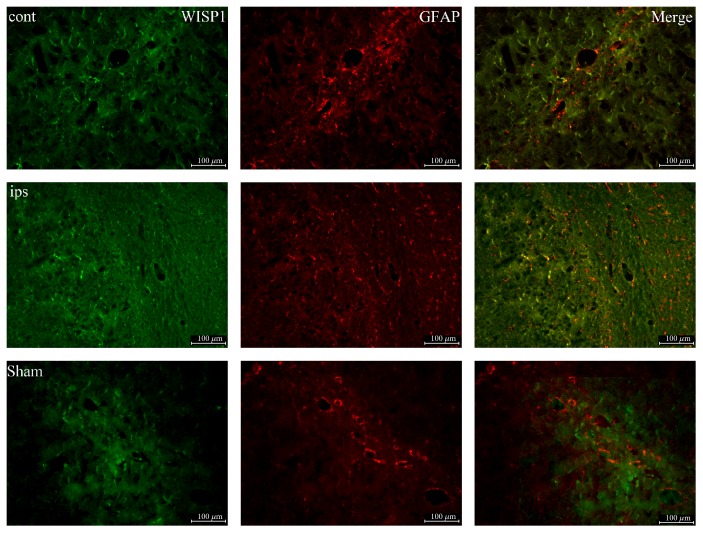
Double-labeling immunofluorescence of WISP1 and GFAP at 3 d after TBI. Green: WISP1; red: GFAP; cont: the control of ipsilateral 3 d post-TBI group; ips: ipsilateral 3 d post-TBI group; sham: sham group; bar = 100 *μ*m.

To investigate the cell type that WISP1 located, double-labeling immunofluorescence was performed. The colocalizations of WISP1 with astrocyte marker GFAP, neuronal marker NeuN, microglia marker Iba1 were examined respectively. Compared with the sham group, WISP1-positive signals decreased slightly in the control lateral brain and reduced significantly in the ipsilateral brain. However, GFAP positive signals increased slightly in the control lateral brain and had a dramatic increase in the ipsilateral brain. There was a little amount colocalization between WISP1 and GFAP in the sham group. However, obvious colocalizations were observed in the brain at 3 d after TBI including ipsilateral and control lateral brain (Figure 2).

**Figure 3:**
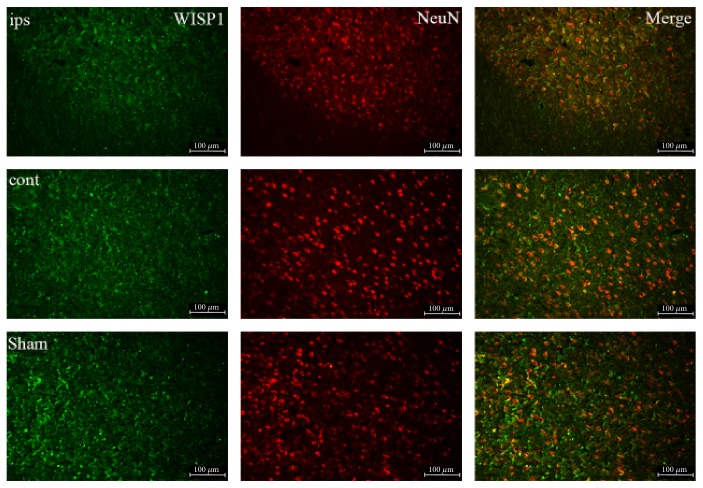
Double-labeling immunofluorescence of WISP1 and NeuN at 3 d after TBI. Green: WISP1; red: NeuN; cont: the control of ipsilateral 3 d post-TBI group; ips: ipsilateral 3 d post-TBI group; sham: sham group; bar = 100 *μ*m.

NeuN-positive signals obviously decreased in the ipsilateral brain and decreased slightly in the control lateral brain compared with the sham group (Figure 3). Double-labeling immunofluorescence demonstrated that there were obvious colocations of WISP1 and NeuN either in ipsilateral or in control lateral brain after TBI at 3 d compared with the sham group.

**Figure 4:**
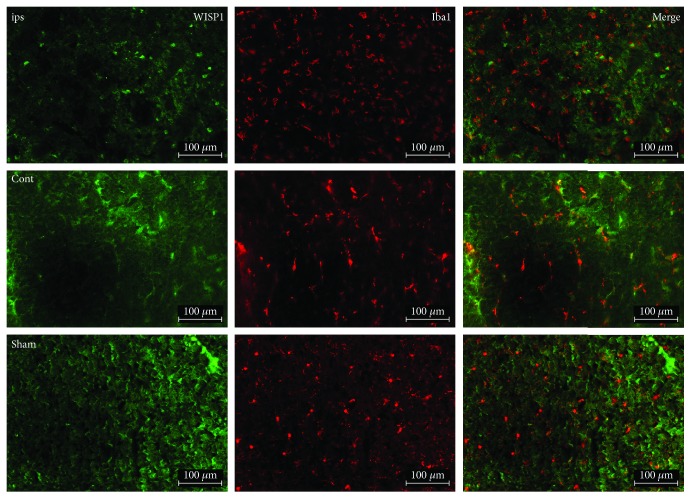
Double-labeling immunofluorescence of WISP1 and Iba1 at 3 d after TBI. Green: WISP1; red: Iba1; cont: the control of ipsilateral 3 d post-TBI group; ips: ipsilateral 3 d post-TBI group; sham: sham group; bar = 100 *μ*m.

At the same time, there was a modicum colocation of WISP1 and Iba1. Compared with the sham group, Iba1-positive signals increased substantially in the ipsilateral brain (Figure 4).

### 3.2. WISP1 and the Relationship between Autophagy and Apoptosis

**Figure 5:**
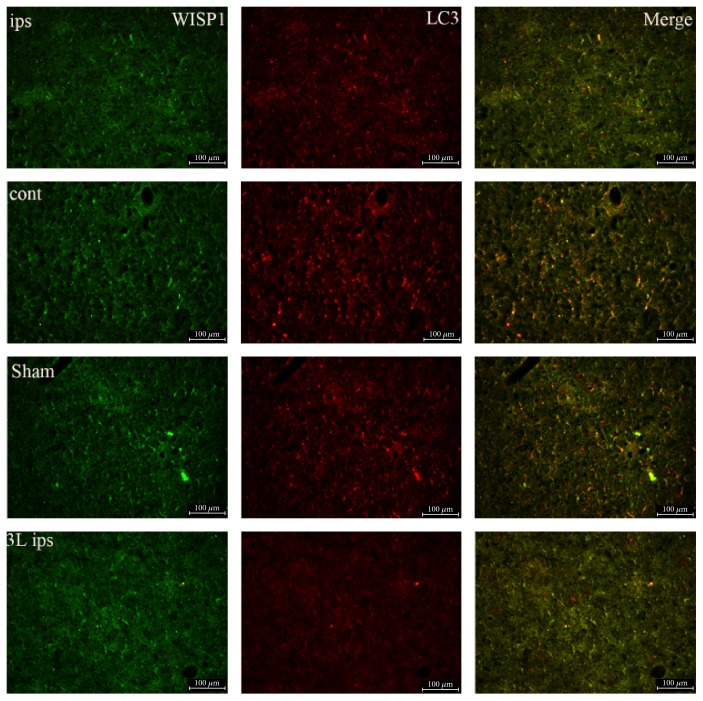
Double-labeling immunofluorescence of WISP1 and LC3 at 3 d after TBI. Green: WISP1; red: LC3; cont: the control of ipsilateral 3 d post-TBI group; ips: ipsilateral 3 d post-TBI group; sham: sham group; 3 L ips: 3 d post-TBI + 1 mM PQQ group; cont: the control of ipsilateral 3 d post-TBI + 1 mM PQQ group. Bar = 100 *μ*m.

Brain injury was often accompanied by the occurrence of apoptosis and autophagy. In the present study, double immunofluorescence was used to detect the colocalization of WISP1 and autophagy marker LC3 and apoptosis marker cleaved caspase-3. Compared with the sham group, LC3-positive signals had a substantial increase in the ipsilateral brain and had no significant change in the control lateral brain after 3 d post-TBI. After treatment with 1 mM PQQ at 3 d post-TBI, WISP1-positive signals rose obviously; LC3 positive signals declined markedly in the ipsilateral brain. Double-labeling immunofluorescence showed that there were a lot of colocalization of WISP1 and LC3 (Figure 5).

**Figure 6:**
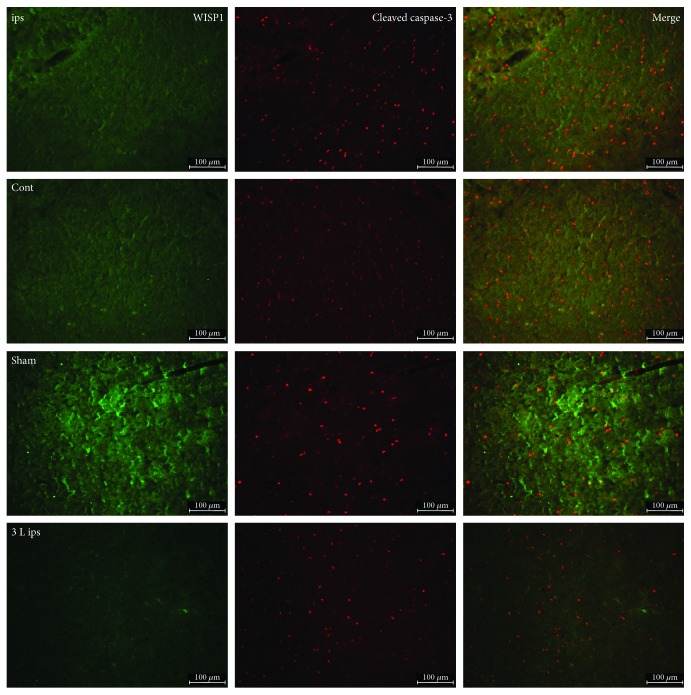
Double-labeling immunofluorescence of WISP1 and cleaved caspase-3 at 3 d after TBI. Green: WISP1; red: cleaved caspase-3; cont: the control of ipsilateral 3 d post-TBI group; ips: ipsilateral 3 d post-TBI group; sham: sham group; 3 L ips: 3 d post-TBI + 1 mM PQQ group; cont: the control of ipsilateral 3 d post-TBI + 1 mM PQQ group. Bar = 100 *μ*m.

After 3 d post-TBI, compared with the sham group, cleaved caspase-3 expression was obviously upregulated in the ipsilateral brain and had no significant change in the control lateral brain. After treatment with 1 mM PQQ at 3 d post-TBI, cleaved caspase-3-positive signals declined obviously in the ipsilateral brain. Double-labeling immunofluorescence showed that there was a little colocalization of WISP1 and cleaved caspase-3 (Figure 6).

### 3.3. The Relationship of WISP1 and *β*-Catenin

**Figure 7:**
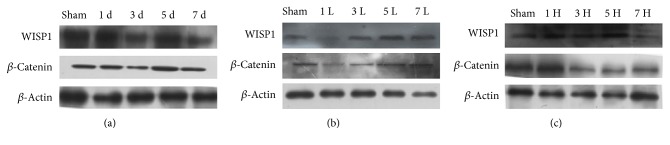
WISP1 and *β*-catenin protein expression (*P* < 0.05, *P* < 0.01). Sham: sham group; 1 d: ipsilateral 1 d post-TBI group; 3 d: ipsilateral 3 d post-TBI group; 5 d: ipsilateral 5 d post-TBI group; 7 d: ipsilateral 7 d post-TBI group; 1 L: 1 d post-TBI + 1 mM PQQ group; 3 L: 3 d post-TBI + 1 mM PQQ group; 5 L: 5 d post-TBI + 1 mM PQQ group; 7 L: 7 d post-TBI + 1 mM PQQ group; 1 H: 1 d post-TBI + 2 mM PQQ group; 3 H: 3 d post-TBI + 2 mM PQQ group; 5 H: 5d post-TBI + 2 mM PQQ group; 7 H: 7 d post-TBI + 2 mM PQQ group.

The expression changes of WISP1 and *β*-catenin protein were performed by Western blot, which indicated that the protein expression trend of WISP1 was similar to that of *β*-catenin at different time points after TBI. The protein expression of WISP1 and *β*-catenin decreased slightly at 1 d after TBI, minimized at 3 d post-TBI and rose again at 5 d after TBI, and slightly reduced at 7 d after TBI compared with the sham group (Figure 7(a)). Meanwhile, the treatment with 1 mM PQQ increased the protein expression of WISP1 and *β*-catenin at 3 d, 5 d, and 7 d post-TBI (Figure 7(b)). When 2 mM PQQ was treated, there was no obvious change trend (Figure 7(c)).

**Figure 8:**
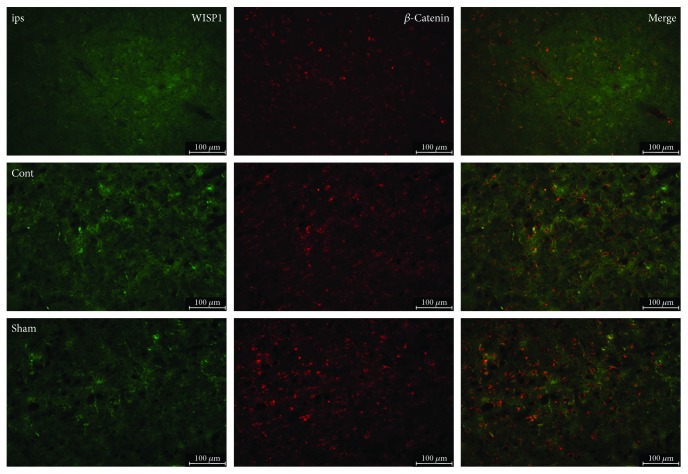
Double-labeling immunofluorescence of WISP1 and *β*-catenin at 3 d after TBI. Green: WISP1; red: *β*-catenin; cont: the control of ipsilateral 3 d post-TBI group; ips: ipsilateral 3 d post-TBI group; sham: sham group; bar = 100 *μ*m.

After 3 d post-TBI, as far as the sham group, WISP1 expression significantly reduced in the ipsilateral brain and slightly reduced in the control brain. Moreover, *β*-catenin-positive signal change trend was similar to that of WISP1. Double-labeling immunofluorescence showed that there was a lot colocalization of WISP1 with *β*-catenin (Figure 8).

**Figure 9:**
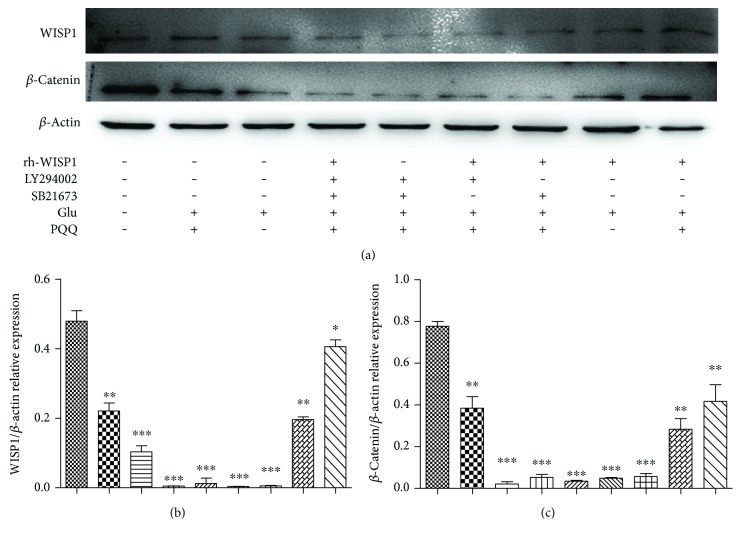
WISP1 and *β*-catenin protein expression after blocking PI3K/GSK-3*β* signaling pathway (^∗^*P* < 0.05, ^∗∗^*P* < 0.01, ^∗∗∗^*P* < 0.001 versus sham).

The PI3K inhibitor LY294002 (10 *μ*M) and GSK-3*β* inhibitor SB21673 (5 *μ*M) with WISP1 (10 ng/ml) were administrated. The experiments showed that the expression of WISP1 reduced with the administration of glutamate and then upregulated after using 50 *μ*M PQQ as well as WISP1 protein. Interestingly, the expression of WISP1 did not increase under the glutamate after blocking the PI3K/GSK-3*β* signaling pathway, just as the *β*-catenin did (Figure 9).

**Figure 10:**
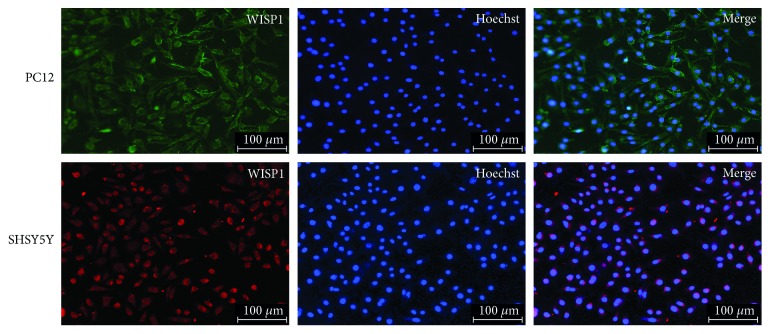
Immunofluorescence of WISP1 in SHSY5Y with PC12 cells. Red or green: WISP1; blue: Hoechst; bar = 100 *μ*m.

Double-labeling immunofluorescence showed that there were a lot of colocalization of WISP1 and neuron in TBI, so SHSY5Y cells and PC12 cells were chosen to study further. Immunofluorescence displayed that WISP1 distributed in the cytoplasm either SHSY5Y cells or PC12 cells (Figure 10).

**Figure 11:**
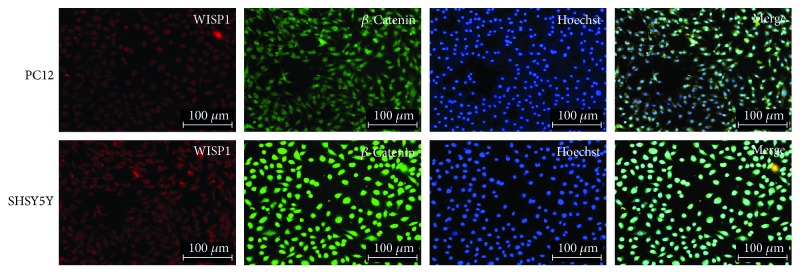
Double-labeling immunofluorescence of WISP1 in SHSY5Y with PC12 cells. Red: WISP1; green: *β*-catenin; blue: Hoechst; bar = 100 *μ*m.

The colocalization of WISP1 and *β*-catenin were detected in SHSY5Y cells or PC12 cells. Immunofluorescence showed that *β*-catenin was distributed in the cytoplasm of SHSY5Y cells or PC12 cells, and there was obvious expression in nuclear of SHSY5Y cells. Double-labeling immunofluorescence showed that WISP1 colocalizated with *β*-catenin in the cytoplasm of SHSY5Y or PC12 cells (Figure 11).

### 3.4. The Effect of PQQ on the Viability of Cells

**Figure 12:**
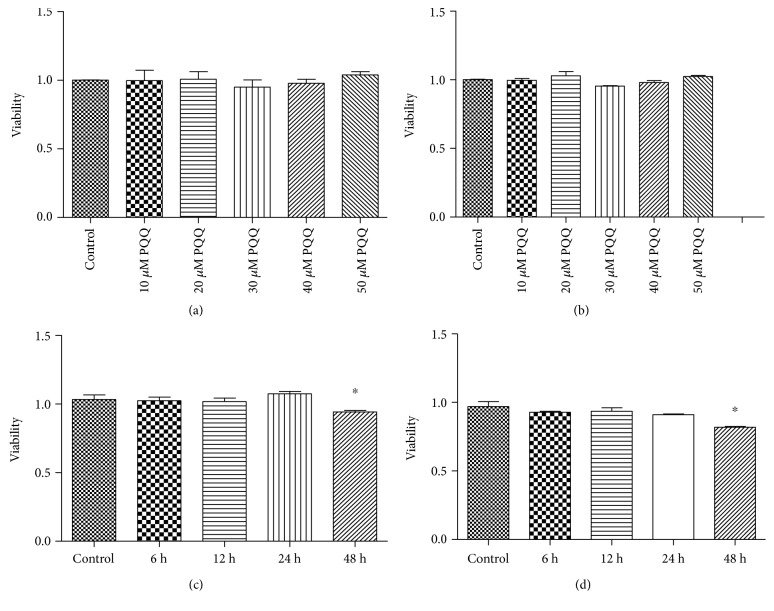
The effect of PQQ on the viability of PC12 and SHSY5Y cells. (a) Control: PC12 cells without PQQ; (b) control: SHSY5Y cells without PQQ; (c) control: PC12 cells without PQQ, ^∗^*P* < 0.05 versus control; (d) control: SHSY5Y cells without PQQ, ^∗^*P* < 0.05 versus control.

The effects of different concentrations or time of PQQ on the activity of PC12 and SHSY5Y cells were performed with CCK-8 assay. The results showed, incubating PC12 cells for 24 h with 10, 20, 30, 40, and 50 *μ*M PQQ, that there was no distinct effect on PC12 cell activity compared with the control group (Figure 12(a)). Meanwhile, the similar results were obtained in SHSY5Y cells. That is, there was no obvious change of cell viability in SHSY5Y cells treated with or without 10, 20, 30, 40, and 50 *μ*M PQQ for 24 h (Figure 12(b)). When 50 *μ*M PQQ was incubated for 6 h, 12 h, 24 h, and 48 h in PC12 cells, there was slight influence on PC12 cell activity at 48 h (Figure 12(c)). At the same time, adopting the same approach in SHSY5Y cells, the activity of SHSY5Y cells was consistent with PC12 cell activity (Figure 12(d)).

**Figure 13:**
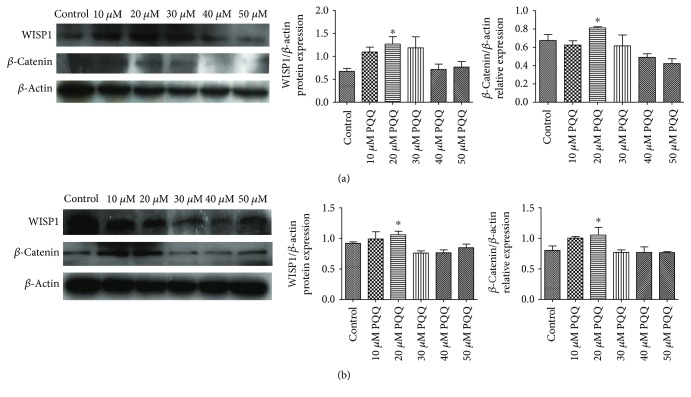
WISP1 and *β*-catenin protein expressions in PC12 cells and SHSY5Y cells with the treatment of different concentrations of PQQ. (a) Control: PC12 cells without PQQ; 10, 20, 30, 40, and 50 *μ*M: different concentrations of PQQ for 24 h; (b) control: SHSY5Y cells without PQQ (^∗^*P* < 0.05).

Western blot was used to detect the protein expression of WISP1 in PC12 or SHSY5Y cells that were treated with different concentrations of PQQ for 24 h. Compared with the control group, the expression of WISP1 increased slightly with 10 *μ*M PQQ, reached the peak in PC12 cells treated with 20 *μ*M PQQ for 24 h, and then other groups decreased gradually. Moreover, *β*-catenin expression in PC12 cells peaked with 20 *μ*M PQQ for 24 h. There was no obvious difference with 10, 30, 40, and 50 *μ*M PQQ (Figure 13(a)). Meanwhile, the similar results were obtained in SHSY5Y cells (Figure 13(b)).

**Figure 14:**
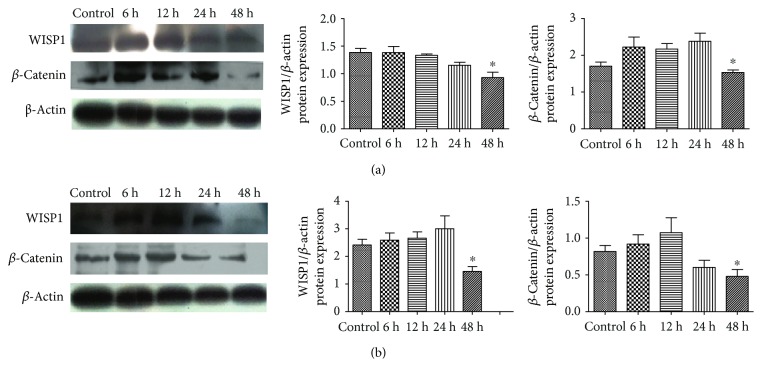
WISP1, *β*-catenin protein expression in PC12 cells, and SHSY5Y cells with the treatment of 50 *μΜ* PQQ at different time. (a) Control: PC12 cells without PQQ; 6 h, 12 h, 24 h, and 48 h: different time points; (b) control: SHSY5Y cells without PQQ (^∗^*P* < 0.05).

50 *μ*M PQQ was used to detect the protein expression of WISP1 in PC12 or SHSY5Y cells at 6, 12, 24, and 48 h. Compared with the control group, results showed that the expression of WISP1 reduced gradually in a time-dependent manner and reached the minimum in PC12 cells treated with 50 *μ*M PQQ for 48 h. *β*-Catenin protein expression in PC12 cells reached the minimum at 48 h and increased slightly at 6 h, 12 h, and 24 h compared with the control group, but it was not significant (*P* > 0.05) (Figure 14(a)). At the same time, the similar results were obtained in SHSY5Y cells. The expression of WISP1 in SHSY5Y cells was similar to that of *β*-catenin in PC12 cells; *β*-catenin protein expression in SHSY5Y cells reached the minimum at 48 h and increased slightly at 6 h and 12 h, but there was no significant difference compared with the control group (*P* > 0.05) (Figure 14(b)).

**Figure 15:**
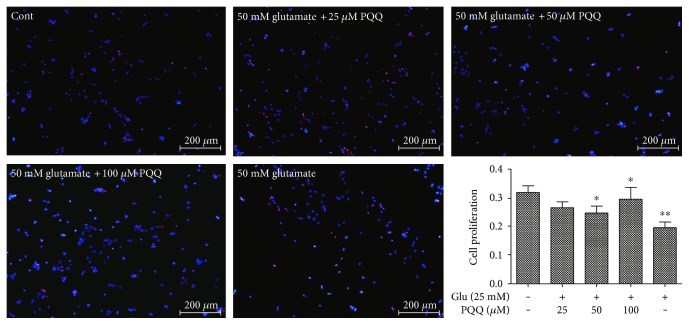
The proliferation of astrocytes with glutamate and PQQ (^∗^*P* < 0.05, ^∗∗^*P* < 0.01).

Prior to adding the glutamate, 25 *μ*M, 50 *μ*M, and 100 *μ*M PQQ were added in the cell medium, respectively. EdU assay results showed that PQQ could promote the proliferation of astrocytes inhibited by glutamate (^∗^*P* < 0.05, ^∗∗^*P* < 0.01) (Figure 15).

**Figure 16:**
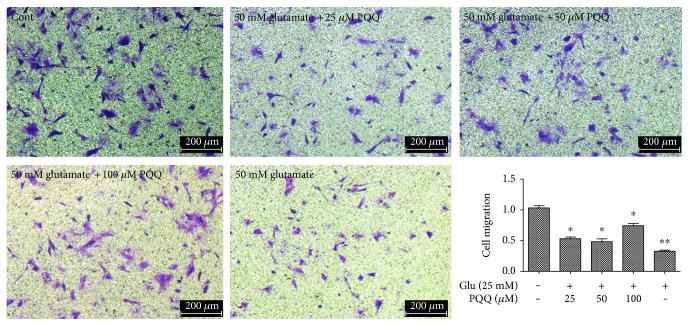
The migration of astrocytes after glutamate and PQQ (^∗^*P* < 0.05, ^∗∗^*P* < 0.01).

Transwell migration assay demonstrated that 5 *μ*M, 50 *μ*M, and 100 *μ*M PQQ could reverse the astrocyte migration inhibited by glutamate (^∗^*P* < 0.05, ^∗∗^*P* < 0.01) (Figure 16).

**Figure 17:**
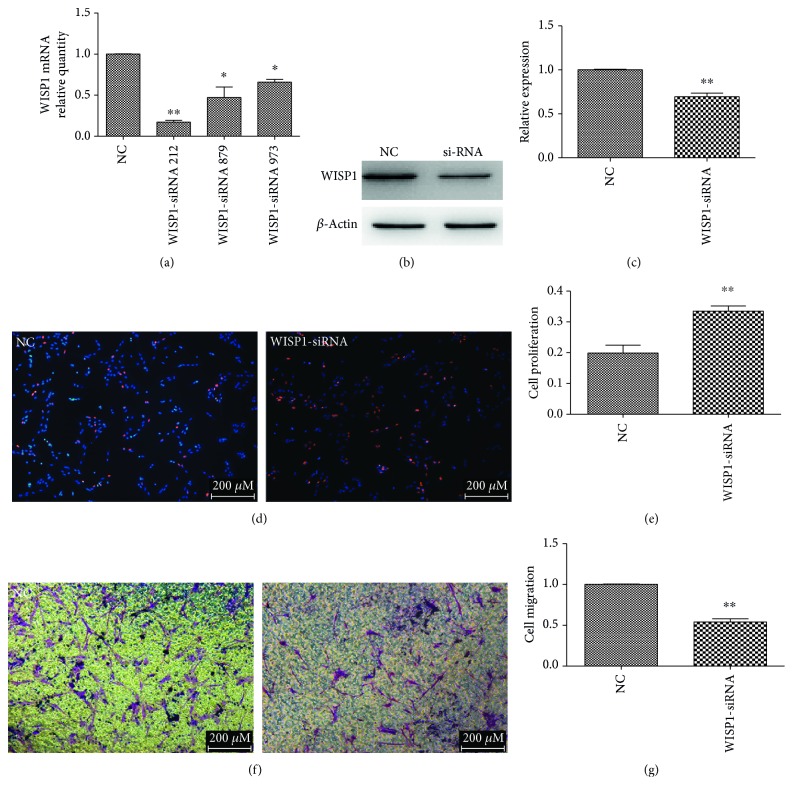
The proliferation and migration of astrocytes after transfected by WISP1 si-RNA. NC: negative control group (^∗^*P* < 0.05). (a) qRT-PCR showed that the expression of WISP1 in astrocytes transfected with WISP1 si-RNA. (b) Western blot showed that WISP1 si-RNA could decreased the expression of WISP1 protein. (c) The statistic graph of (b). (d) Proliferation of astrocytes transfected with WISP1 si-RNA compared with the normal group. (e) The statistic graph of (d) (^∗∗^*P* < 0.01). (f) The migration of astrocytes transfected with WISP1 si-RNA compared with the normal group. (g) The statistic graph of (f) (^∗∗^*P* < 0.01).

To observe the function of WISP1, WISP1 si-RNA was transfected in the astrocytes. Compared with negative control group, the expression of WISP1 obviously declined after being transfected with WISP1 si-RNA for 48 h, which suggested that WISP1 si-RNA was efficient (^∗^*P* < 0.05, ^∗∗^*P* < 0.01) (Figures 17(a), 17(b), and 17(c)).

The expression of WISP1 was silenced, and the EdU assay was performed. WISP1 could increase the positive signals of astrocytes (^∗∗^*P* < 0.01) (Figures 17(d) and 17(e)). Similarly, to determine whether WISP1 plays an important role in cell migration, transwell migration assay was used to detect the migration of astrocytes; the results show that migrated cell numbers were significantly increased following interference expression of WISP1 (^∗∗^*P* < 0.01) (Figures 17(f) and 17(g)).

## 4. Discussion

Traumatic brain injury (TBI) is mainly caused by an external mechanical force, which can be divided into primary and secondary damage. TBI can be classified based on the severity or mechanism of injury, as well as other features. In most cases, TBI leads to higher mortality rates and disability; moreover, TBI could cause physical, pathological, and behavioral effects. Therefore, more and more people attracted general attention. Protecting neurons are the main methods to treat cerebral trauma, and the treatment goals are focused in the cell death program targets. However, there is not an effective method to treat TBI so far.

WISP1 is a family of secreted proteins and regulate various developmental processes. Wnt1 proteins combine the cell membrane receptor by autocrine or paracrine effects, activate intracellular signaling pathways, regulate target gene expression, and play an important role on cell proliferation, polarity and differentiation, and migration. WISP1 secreted Wnt1-induced protein 1, also known as CCN4, cognating connective tissue growth factor (CTGF) [2]. WISP1 was later associated with neoplastic growth in the gastrointestinal tract [25]. In the present study, WISP1 mRNA expression was detected by qRT-PCR after brain injury. Compared with the sham group, WISP1 mRNA expression reached to the minimum at 3 d and then recovered at 5 d after TBI. Meanwhile, the expression of WISP1 mRNA increased with 1 mM or 2 mM PQQ at different time post-TBI, but the effect of 1 mM PQQ was obvious to the 2 mM PQQ, which might be the concentration of 1 mM PQQ in animals that can reach the optimum serum concentration in TBI.

Astrocyte activation is a common reaction in the central nervous system under many pathophysiological situations, which assume astrocyte cell hypertrophy, swelling, protuberance extension, and glial fibrillary acidic protein expression increase [26, 27]. Astrocytes began to activate after TBI and may even cause several neurodegenerative diseases. In the paper, double-labeling immunofluorescence was performed to detect the cell type that WISP1 located in the brain. Results indicated WISP1 located mainly in the neuron of the brain through the colocalization of WISP1 with astrocyte marker GFAP, neuronal marker NeuN, and microglia marker Iba1, respectively.

After traumatic brain injury, there would be different levels of autophagy. Autophagy has three different categories termed microautophagy, macroautophagy, and chaperone-mediated autophagy [28]. Autophagy is a eukaryotic cell metabolic process, autophagy with disease-linked, is gradually increasing in the recent years. Autophagy is involved in pathogenesis of neuronal injury and death including mitochondrial damage, activating inflammation, oxidative free radicals, and caspase activation [29]. Apart from cell death which was caused by TBI, autophagic cell death and apoptosis occupy a considerable proportion, autophagy protects damaged cells and also exacerbates cell damage, which mainly depend on the role of autophagy after injury and stage. Autophagy is involved in regulation of cell survival after death in traumatic brain injury, and as a result, autophagy is important to brain injury and nerve injury and repair [30]. Double immunofluorescence of WISP1 and autophagy marker LC3 showed that WISP1 and LC3 had a lot of colocalization at 3 d post-TBI. After treatment with 1 mM PQQ, WISP1-positive signals rose obviously; LC3-positive signals declined markedly in the ipsilateral brain. All these data demonstrated that PQQ may have a protective role on autophagy. In addition, apoptosis is believed to be a significant contributor to the pathogenesis of a variety of disorders after TBI. For example, in the brains of patients with Alzheimer's disease, apoptotic DNA fragmentation [31] and caspase activation have been observed [32]. Caspase-3 activation is relevant to the apoptosis of neurons after various types of damage. In this study, double-labeling immunofluorescence showed that WISP1 colocalizated with cleaved caspase partly. Cleaved caspase-3 expression was obviously upregulated in the ipsilateral brain after 3 d post-TBI. After treatment with 1 mM PQQ, cleaved caspase-3-positive signals declined obviously, which reminded that WISP1 might not be involved directly in the process of apoptosis.

WISP1 and *β*-catenin act as new targets investigated in recent years, which can promote tissues proliferation as well as various cell repair. In regard to the canonical pathways of Wnt1 [33], WISP1 can block phosphorylation of *β*-catenin in neurons that may be mediated through the inhibition of GSK-3*β* which prevent *β*-catenin phosphorylation [33]. WISP1 also can block GSK-3*β* activity in other cell systems such as cardiac cells [12, 34]. In neurons, WISP1 through a PI3K-mediated pathway promotes the nucleus expression of *β*-catenin [4]. In addition, WISP1 expression is governed by *β*-catenin activity and WISP1 regulates its own expression through the ability of WISP1 to control *β*-catenin phosphorylation and nuclear translocation [4]. Western blot showed that the protein expression of WISP1 and *β*-catenin minimized at 3 d and rose again at 5 d after TBI compared with the sham group. Meanwhile, the treatment with 1 mM or 2 mM PQQ increased the protein expression of WISP1 and *β*-catenin correspondingly. All these results demonstrated that WISP1 may modulate the protein expression of *β*-catenin.

PQQ, also known as methoxatin, has been detected in a wide variety of foods and other sources. PQQ, as an important antioxidant and nutrient, has also begun to focus on nutriology and pharmacology. PQQ belongs to vitamin B [21]. PQQ has many other pharmacological effects, including anti-inflammatory, hepatoprotective, heart protection, and antioxidizing effect [22, 35, 36]. PQQ has a neuroprotective function and a very good effect on the epilepsy model by PI3K/AKT signaling pathway [37]. Although the role of PQQ as a vitamin in animal or human nutrition is controversial, accumulating evidence suggest that PQQ plays important roles on cell protection. In the experiment, we used different concentrations of PQQ to treat SHSY5Y or PC12 cells; results showed that PQQ had no toxicity on the cell lines. Similarly, PQQ had no significant effect on the animal without TBI. In subsequent experiments, in addition, PQQ may protect cells through influencing the expression of WISP1.
